# Association of laryngopharyngeal reflux with chronic rhinosinusitis prevalence in adults: A systematic review and meta-analysis

**DOI:** 10.17305/bb.2025.13354

**Published:** 2025-12-25

**Authors:** Jingda Xu, Min Chen, Gang Chen, Ting Lou, Long Xu

**Affiliations:** 1 Faculty of Chinese Medicine, Macau University of Science and Technology, Macau, China; 2 Department of Otolaryngology, Zhuhai Hospital of Integrated Traditional Chinese and Western Medicine, Zhuhai, China; 3 Department of Physical Examination, Shenzhen University General Hospital, Shenzhen, China; 4 Department of Gastroenterology and Hepatology, Shenzhen University General Hospital, Shenzhen, China

**Keywords:** Laryngopharyngeal reflux, chronic rhinosinusitis, association, meta-analysis

## Abstract

Laryngopharyngeal reflux (LPR) has been implicated in the pathogenesis of chronic rhinosinusitis (CRS), but the evidence from individual studies remains inconsistent. This meta-analysis aims to clarify the association between LPR and CRS in adults. We systematically searched PubMed, Embase, Web of Science, CNKI, and Wanfang for observational studies that evaluate the relationship between LPR and CRS in adult populations. Heterogeneity among studies was assessed using the Cochrane *Q* test and the *I*^2^ statistic. Odds ratios (ORs) and 95% confidence intervals (CIs) were pooled using a random-effects model to account for heterogeneity. A total of eight cross-sectional studies involving 3456 participants were included in the analysis. The results indicated a significant association between LPR and a higher prevalence of CRS in adults (OR = 4.77, 95% CI: 2.51–9.07; *P* < 0.001; *I*^2^ ═ 63%). Sensitivity analysis restricted to high-quality studies (Newcastle–Ottawa Scale score ≥ 7) produced similar results with no observed heterogeneity (OR = 5.98, 95% CI: 3.60–9.92; *I*^2^ ═ 0%). Exploratory subgroup analyses suggested a stronger association in studies with smaller sample sizes and when both LPR and CRS were diagnosed using objective methods. No significant evidence of publication bias was detected through Egger’s test (*P* ═ 0.35); however, this analysis was underpowered and should be interpreted cautiously in the context of the small-study effect. In conclusion, LPR may be associated with an increased prevalence of CRS in adults, especially when both conditions are diagnosed using objective criteria. Further prospective studies are needed to confirm this association and explore the underlying mechanisms.

## Introduction

Chronic rhinosinusitis (CRS) is a prevalent and often debilitating inflammatory disorder of the nasal and paranasal sinuses that lasts for a minimum of 12 weeks [1–3]. It affects approximately 5%–15% of the adult population globally, significantly impacting quality of life, daily functioning, and healthcare costs [4, 5]. Patients commonly experience persistent nasal obstruction, rhinorrhea, facial pain or pressure, and impaired olfaction, collectively diminishing productivity and overall well-being [1]. While established risk factors for CRS include allergic rhinitis, asthma, smoking, occupational exposures, and anatomical variations, the precise etiology remains incompletely understood in many instances [6]. Therefore, identifying additional potentially modifiable factors related to CRS is crucial for enhancing prevention, early detection, and treatment outcomes.

Laryngopharyngeal reflux (LPR) is characterized by the retrograde flow of gastric or duodenal contents into the larynx, pharynx, and upper airway structures [7]. Unlike typical gastroesophageal reflux disease (GERD), LPR often occurs when individuals are upright and may present without heartburn or regurgitation [8]. Diagnosis of LPR can be achieved through symptom-based tools such as the reflux symptom index (RSI), endoscopic findings assessed by the reflux finding score (RFS), or objective methods, including 24-h pH or impedance–pH monitoring and detection of pepsin in upper airway secretions [9, 10]. The prevalence of LPR in adults is estimated to range from 10% to 30%, depending on the diagnostic approach and population studied [11]. Biologically, refluxed gastric acid, pepsin, and bile salts may damage sinonasal mucosa, impair mucociliary clearance, and promote chronic inflammation, potentially contributing to the development or persistence of CRS [12, 13]. Despite several observational studies over the past two decades investigating the relationship between LPR and CRS, findings have been inconsistent, likely attributable to variations in study design, populations, diagnostic methods, and analytic adjustments [14–21]. To address these uncertainties, we conducted a systematic review and meta-analysis of observational studies to quantitatively assess the association between LPR and CRS in adults and to explore potential sources of heterogeneity across studies.

## Materials and methods

This study adhered to the Preferred Reporting Items for Systematic Reviews and Meta-Analyses (PRISMA) 2020 guidelines [22, 23] and the Cochrane Handbook for systematic reviews and meta-analyses [24], encompassing study design, data collection, statistical methods, and result interpretation. The protocol for the meta-analysis has been registered at International Prospective Register of Systematic Reviews (PROSPERO) under the identifier: CRD420251156445.

### Database search

To identify relevant studies for this meta-analysis, we searched the PubMed, Embase, Web of Science, Wanfang, and China National Knowledge Infrastructure (CNKI) databases using a comprehensive array of search terms. This included the combined terms: (1) “laryngopharyngeal reflux” OR “LPR” OR “gastro-pharyngeal reflux” OR “gastropharyngeal reflux” OR “GPR” OR “extraesophageal reflux” OR “extra-oesophageal reflux” OR “supraesophageal reflux”; and (2) “chronic rhinosinusitis” OR “chronic sinusitis” OR “sinusitis” OR “CRS”. The search was restricted to human studies and included only full-length articles published in English or Chinese in peer-reviewed journals. Additionally, we manually reviewed the references of related original and review articles to identify further pertinent studies. The search encompassed all records from database inception until August 12, 2025. The complete search strategy for each database is detailed in Supplemental File 1. Grey literature sources were excluded due to their lack of standardized diagnostic definitions for LPR or CRS, insufficient extractable data, and the absence of peer-review, which could compromise methodological consistency and reliability of the results.

### Study eligibility criteria

We applied the PICOS framework to define our inclusion criteria:

- **P (patients)**: Adults (≥18 years) from any clinical or community setting.
- **I (exposure)**: LPR diagnosed according to the criteria established in the original studies, utilizing recognized symptoms, clinical, endoscopic, or instrumental methods.
- **C (comparison)**: Participants without LPR.
- **O (outcome)**: Prevalence of CRS in participants with LPR compared to those without LPR, with CRS diagnosis aligning with the criteria from the original studies.
- **S (study design)**: Observational studies, including cohort studies, case-control studies, and cross-sectional studies reporting comparative data between LPR and non-LPR groups.

We excluded studies conducted exclusively with children (<18 years), those focusing solely on patients with LPR or CRS, interventional randomized controlled trials (RCTs) lacking data on the LPR–CRS association, reviews, meta-analyses, case series, case reports, editorials, studies lacking clear definitions for LPR or CRS, reports without a comparison group or sufficient data for estimating or converting effect measures, duplicate or overlapping cohorts (retaining only the most comprehensive or recent report), and laboratory, animal, or *in vitro* studies.

### Study quality evaluation

Two authors independently carried out the literature search, study selection, quality assessment, and data extraction. Discrepancies were resolved through discussion with the corresponding author. Study quality was evaluated using a modified version of the Newcastle–Ottawa Scale (NOS) adapted for cross-sectional studies [25], as previously applied in meta-analyses [26, 27]. The evaluation rubric included selection (4 items), comparability (2 items), and outcome (3 items), with a maximum score of 9 points. Specific items and scoring criteria are detailed in Supplemental File 2. Studies scoring ≥7 were deemed high quality.

### Data collection

Data collected for analysis encompassed study details (author, year, study country, and design), participant characteristics (sample size, mean age, and sex distribution), methods for diagnosing LPR and the number of patients diagnosed, methods for diagnosing CRS and the number of patients diagnosed, and covariates adjusted in the analysis of the LPR–CRS association.

### Statistical analysis

The association between LPR and the prevalence of CRS in adults was summarized as odds ratios (ORs) along with corresponding 95% confidence intervals (CIs) [24]. ORs and standard errors were either directly extracted or calculated from 95% CIs or *P* values, subsequently log-transformed to stabilize variance and normalize data [24]. If multiple ORs were reported from different models, the model with the most comprehensive adjustment was utilized. Heterogeneity was assessed using the Cochrane *Q* test and *I*^2^ statistic [28], with a *P* value < 0.10 indicating significant heterogeneity and *I*^2^ values of <25%, 25%–75%, and > 75% signifying low, moderate, and high heterogeneity, respectively. A random-effects model was employed for data pooling, accounting for potential heterogeneity among studies [24]. Primary analyses utilized the DerSimonian–Laird (DL) method. Given the limited number of included studies (*k* ═ 8), a sensitivity analysis was conducted using the restricted maximum likelihood (REML) random-effects model with Hartung–Knapp–Sidik–Jonkman adjustment (HKSJ), which provides more reliable CIs in small-sample meta-analyses [24]. The REML–HKSJ estimate closely aligned with the DL result and was regarded as the more conservative measure of uncertainty, with DL results presented primarily for comparability with previous studies. Additionally, τ^2^ and 95% prediction intervals (PIs) were calculated [24]. Sensitivity analyses were performed by sequentially removing one study at a time. For the primary outcome, predefined subgroup analyses were executed based on study country (Western vs Asian countries), sample size, mean ages, proportions of men, diagnostic methods for LPR (self-report based on symptoms vs objective evaluation), diagnostic methods for CRS (symptom-based vs objective evidence), and analytic models (univariate vs multivariate). Publication bias was assessed using funnel plots and visual inspection for asymmetry, supplemented by Egger’s test [29]. All analyses were conducted using RevMan (Version 5.3; Cochrane Collaboration, Oxford, UK) and Stata (Version 17.0; Stata Corporation, College Station, TX, USA).

## Results

### Study inclusion

**Figure 1.**
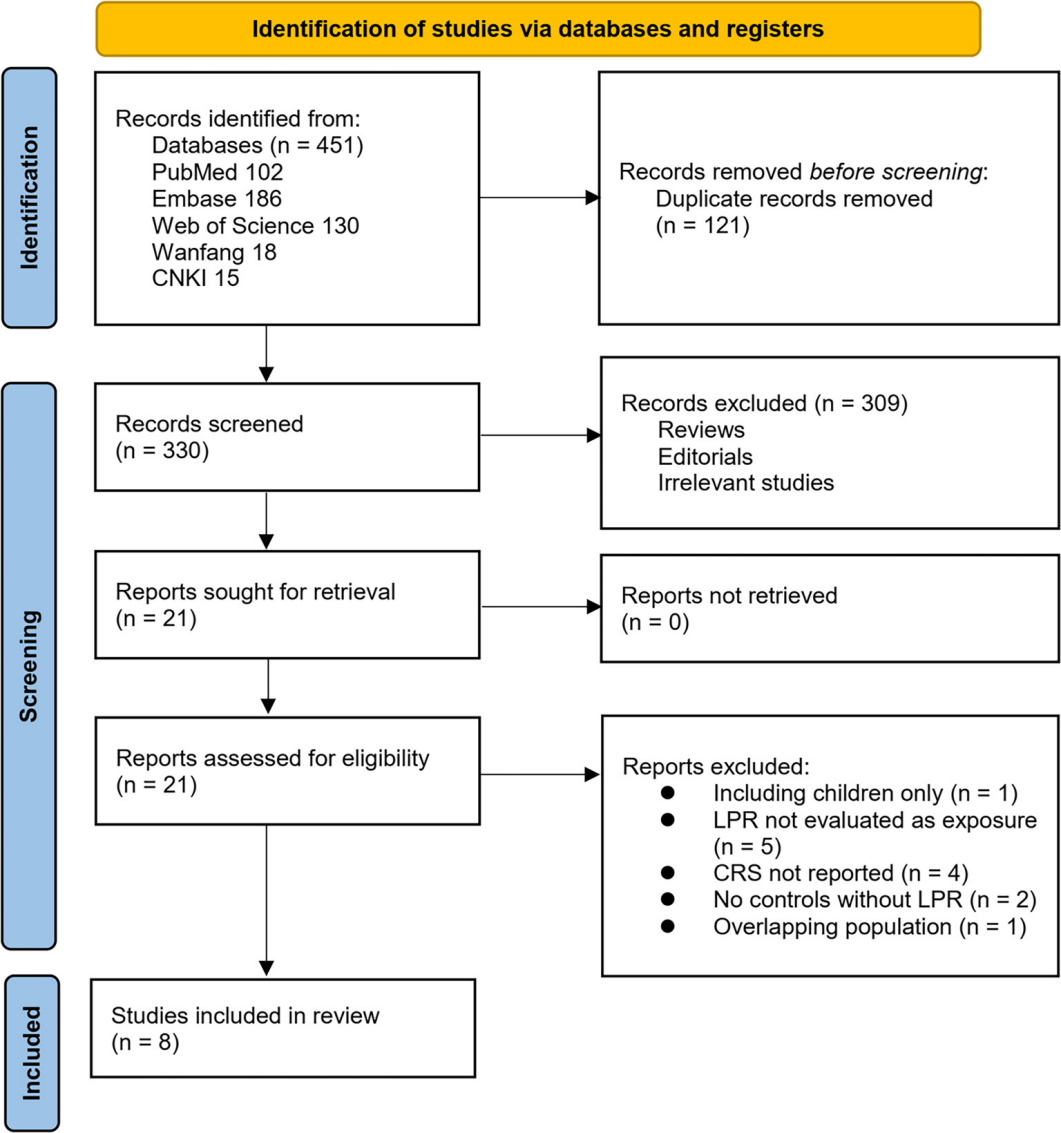
Flowchart of database search and study inclusion.

The study selection process is illustrated in Figure 1. Initially, we identified 451 records from the five databases. Following the removal of 121 duplicates, 330 articles were screened by title and abstract. Of these, 309 were excluded for not aligning with the aims of the meta-analysis. The full texts of the remaining 21 articles were reviewed by two independent authors, and 13 were excluded for various reasons as detailed in Figure 1. Ultimately, eight studies were included in the quantitative analysis [14–21].

**Table 1:** Characteristics of the included studies

**Study**	**Country**	**Design**	**Participant characteristics**	**No. of participants**	**Mean age (years)**	**Men (%)**	**Methods for the diagnosis of LPR**	**No. of patients with LPR**	**Methods for the diagnosis of CRS**	**No. of patients with CRS**	**Variables adjusted**
Ulualp, 1999	USA	CS	Patients with otolaryngologic symptoms and findings, and healthy controls	101	46.6	53.5	24-h triple-sensor pH monitoring	43	Symptoms + CT staging + prior treatment failure	18	None
DelGaudio, 2005	USA	CS	Adults with refractory CRS after ESS vs controls (successful ESS and non-CRS)	68	47.6	47.1	24-h triple-sensor pH monitoring (nasopharynx, UES, esophagus)	35	History of ESS + persistent symptoms + endoscopic inflammation (EPOS-like criteria)	38	None
Jecker, 2006	Germany	CS	Patients with recurrent CRS after surgery and healthy volunteers	40	36.9	52.5	24-h triple-sensor pH monitoring	13	History + CT + revision surgery confirmation	20	None
Pasic, 2007	USA	CS	Community-dwelling adults (≥18 years) recruited from public venues (hospitals, expos, colleges)	1878	48.5	40.0	Symptom-based (self-reported LPR symptoms)	849	Symptom-based (self-reported nasal congestion/drainage, sinus pain, or medication use)	1333	None
Wang, 2017	China	CS	49 CRS patients (23 CRSwNP, 26 CRSsNP) and 9 normal controls from otolaryngology department	58	39.0	53.4	Pepsin A detection in nasal secretions/tissues (ELISA/Western blot) + RSI questionnaire	35	EPOS 2012 criteria (symptoms + endoscopy + CT)	49	None
Li, 2017	China	CS	Patients undergoing nasal surgery: CRSwNP, CRSsNP, and controls with anatomical abnormalities	46	43.7	58.7	Pepsin detection in nasal tissue by immunohistochemistry	25	Clinical diagnosis + CT (Lund-Mackay score) + pathological confirmation	35	None
Bergqvist, 2023	Sweden	CS	Random population sample aged 50-64 years	1111	58.0	50.0	Self-reported LPR symptoms	109	EPOS criteria	58	Age, sex, BMI, educational level, smoking, and asthma
Shen, 2025	China	CS	Hospitalized CRS patients and healthy volunteers recruited during routine physical examinations	154	41.2	57.1	RSI >13 and/or RFS >7; pepsin >75 ng/mL in nasal secretion (ELISA)	55	EPOS 2020 criteria	104	None

Abbreviations: CS: Cross-sectional study; ESS: Endoscopic sinus surgery; UES: Upper esophageal sphincter; CT: Computed tomography; EPOS: European Position Paper on Rhinosinusitis and Nasal Polyps; CRS: Chronic rhinosinusitis; CRSwNP: Chronic rhinosinusitis with nasal polyps; CRSsNP: Chronic rhinosinusitis without nasal polyps; LPR: Laryngopharyngeal reflux; RSI: Reflux symptom index; RFS: Reflux finding score; ELISA: Enzyme-linked immunosorbent assay; BMI: Body mass index.

### Summary of study characteristics

Table 1 summarizes the characteristics of the eight studies included in this meta-analysis, all of which employed a cross-sectional design and were published between 1999 and 2025. These studies were conducted in the United States, Germany, China, and Sweden, and the study populations varied, including otolaryngology patients with or without CRS, community-dwelling adults, and random population samples. The total sample size ranged from 40 to 1878 participants per study, culminating in a total of 3456 patients included in the meta-analysis. The mean age of participants, where reported, ranged from 36.9 to 58.0 years, with the proportion of men varying from 40.0% to 58.7%. LPR was diagnosed using various methods, including 24-h triple-sensor pH monitoring [14–16], self-reported symptoms [17, 20], pepsin detection in nasal secretions or tissues combined with RSI [18, 19], and RSI/RFS criteria [21]. CRS was identified using definitions based on the European Position Paper on Rhinosinusitis and Nasal Polyps (EPOS) guidelines [19–21] or through clinical history, computed tomography (CT) imaging, or confirmation by revision surgery [14–16, 18]. The study by Pasic (2007) utilized symptom-based self-reporting [17]. Most studies provided unadjusted data, with only one study [20] reporting adjusted estimates for potential confounders, including age, sex, body mass index (BMI), educational level, smoking, and asthma. Study quality was assessed using the NOS (Table 2), with total scores ranging from 6 to 8, indicating moderate to high methodological quality. Most studies performed well in the selection and comparability domains but lacked adjustments for confounders other than age and sex [14–19, 21].

**Table 2:** Study quality evaluation via the Newcastle-Ottawa Scale

**Studies**	**Adequate definition of cases**	**Representativeness of cases**	**Selection of controls**	**Definition of controls**	**Control for age and sex**	**Control for other confounders**	**Exposure ascertainment**	**Same methods for events ascertainment**	**Non-response rates**	**Total**
Ulualp, 1999	1	1	1	1	0	0	1	1	1	7
DelGaudio, 2005	1	1	1	1	0	0	1	1	1	7
Jecker, 2006	1	1	1	1	0	0	1	1	1	7
Pasic, 2007	1	1	1	1	0	0	0	1	1	6
Wang, 2017	1	1	1	1	0	0	1	1	1	7
Li, 2017	1	1	1	1	0	0	1	1	1	7
Bergqvist, 2023	1	1	1	1	1	1	0	1	1	8
Shen, 2025	1	1	1	1	0	0	1	1	1	7

### Association between LPR and CRS

**Figure 2.**
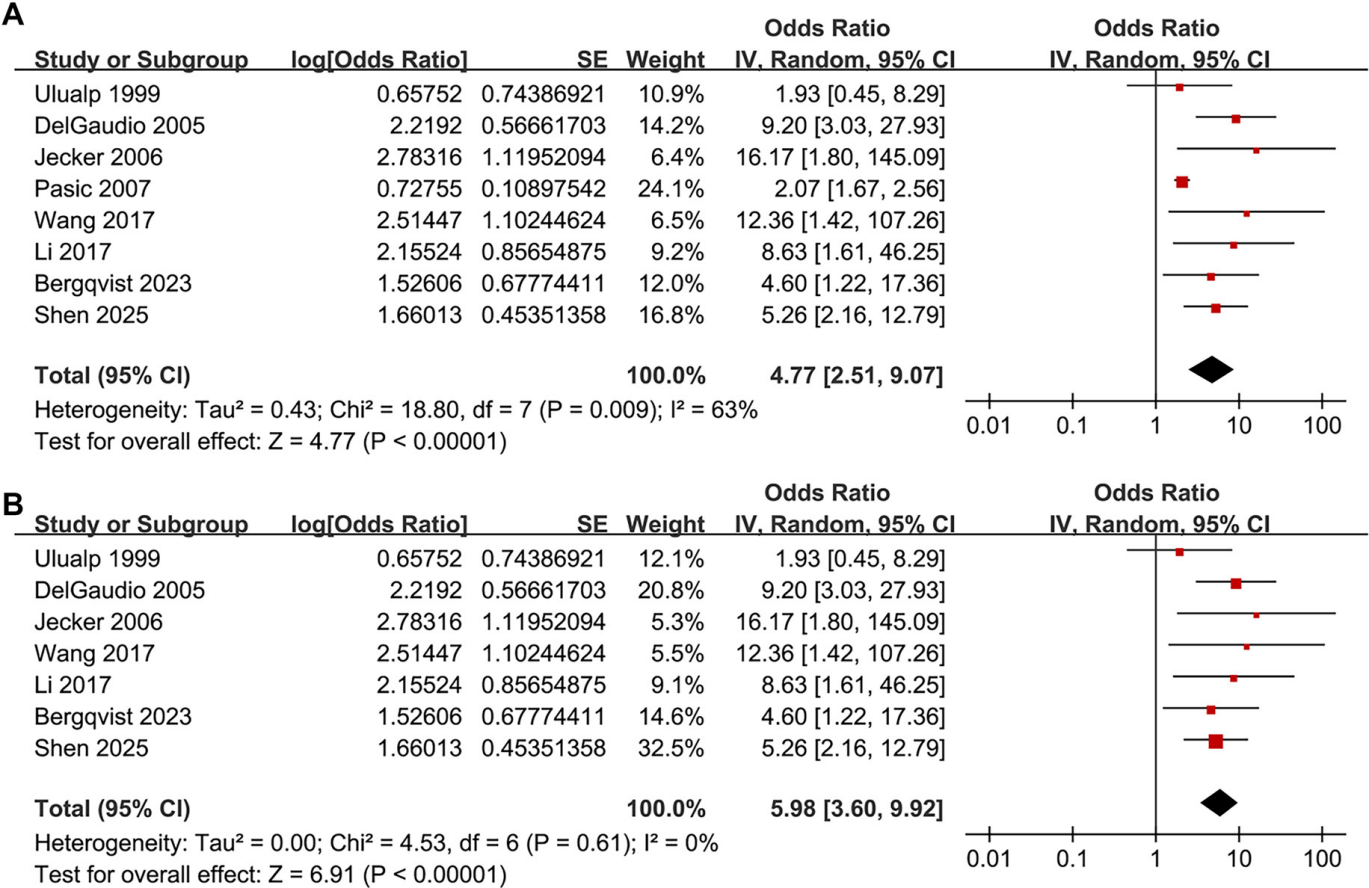
**Association between LPR and CRS in adults—forest plots.** (A) Primary random-effects meta-analysis including all eight eligible cross-sectional studies (total *n* ═ 3456), showing a significant association between LPR and higher CRS prevalence (pooled OR = 4.77, 95% CI 2.51–9.07) with moderate between-study heterogeneity (τ^2^ ═ 0.43; *I*^2^ ═ 63%; Cochran Q *P* ═ 0.009); (B) Sensitivity analysis restricted to high-quality studies (modified NOS ≥ 7), yielding a comparable but more precise estimate (pooled OR = 5.98, 95% CI 3.60–9.92) and no detectable heterogeneity (τ^2^ ═ 0.00; *I*^2^ ═ 0%; Cochran Q *P* ═ 0.61). For each study, squares represent study-specific ORs and are sized proportional to inverse-variance weight; horizontal lines indicate 95% CIs. Diamonds denote pooled effects from an inverse-variance random-effects model (DerSimonian–Laird). The vertical line marks no association (OR = 1), and the *x*-axis is on a logarithmic scale (OR > 1 indicates higher CRS prevalence among participants with LPR). Abbreviations: LPR: Laryngopharyngeal reflux; CRS: Chronic rhinosinusitis; OR: Odds ratio; CI: Confidence interval; Q: Cochran’s Q; NOS: Newcastle–Ottawa Scale.

**Figure 3.**
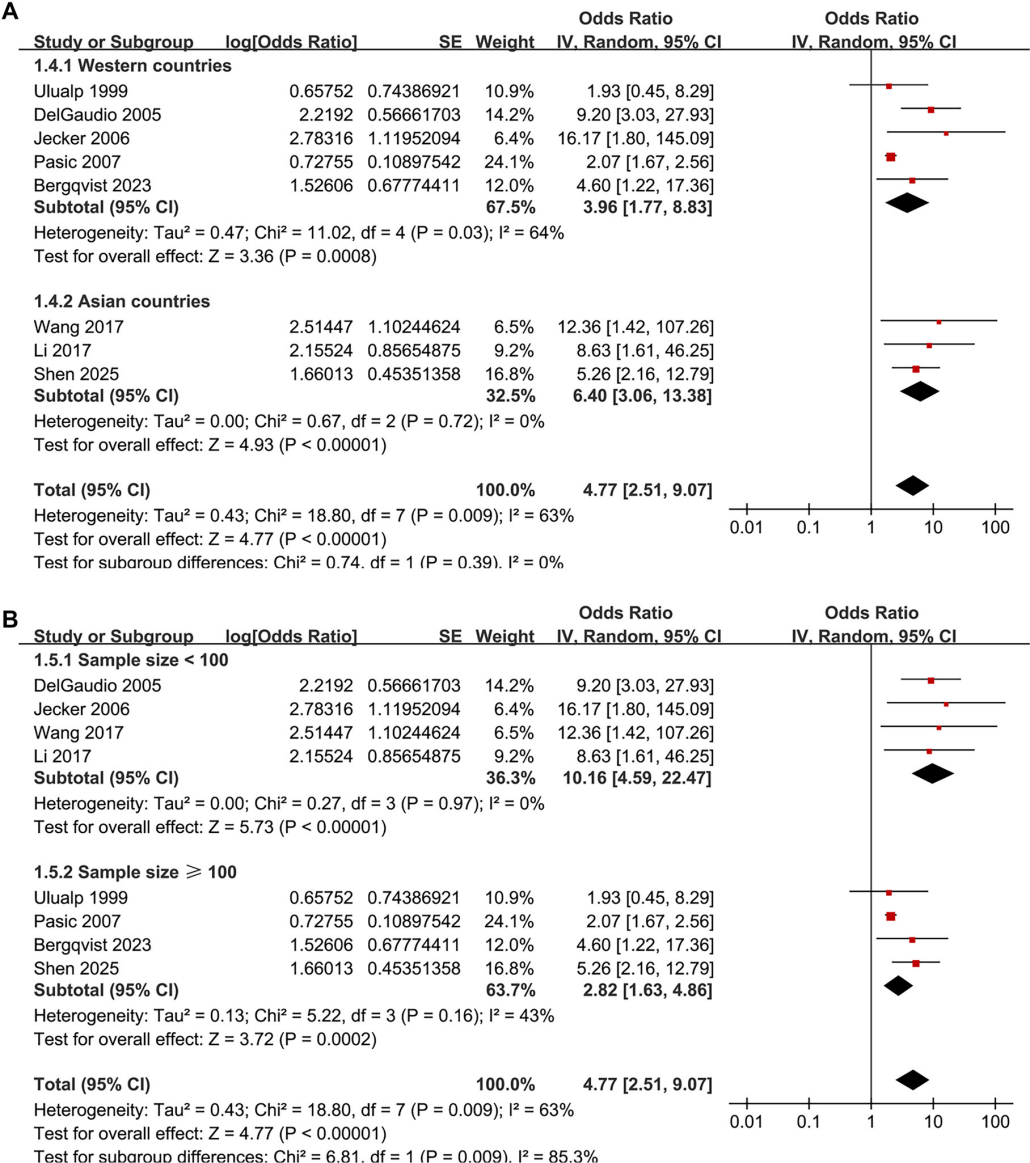
**Subgroup forest plots for the association between LPR and CRS in adults.** (A) Subgroup analysis by study region (Western vs Asian countries). Pooled odds ratios (ORs) were OR = 3.96 (95% CI 1.77–8.83) for Western studies and OR = 6.40 (95% CI 3.06–13.38) for Asian studies, with no statistically significant between-subgroup difference (test for subgroup differences *P* ═ 0.39); (B) Subgroup analysis by study sample size (<100 vs ≥100 participants). Smaller studies reported a larger pooled association (OR = 10.16, 95% CI 4.59–22.47) than larger studies (OR = 2.82, 95% CI 1.63–4.86), with a significant between-subgroup difference (*P* ═ 0.009), consistent with possible small-study effects. Squares represent study-specific ORs (size proportional to inverse-variance weight) with horizontal lines indicating 95% confidence intervals; diamonds indicate pooled effects within each subgroup and overall. The vertical line denotes no association (OR = 1), and the *x*-axis is logarithmic. Abbreviations: LPR: Laryngopharyngeal reflux; CRS: Chronic rhinosinusitis; OR: Odds ratio; CI: Confidence interval.

**Figure 4.**
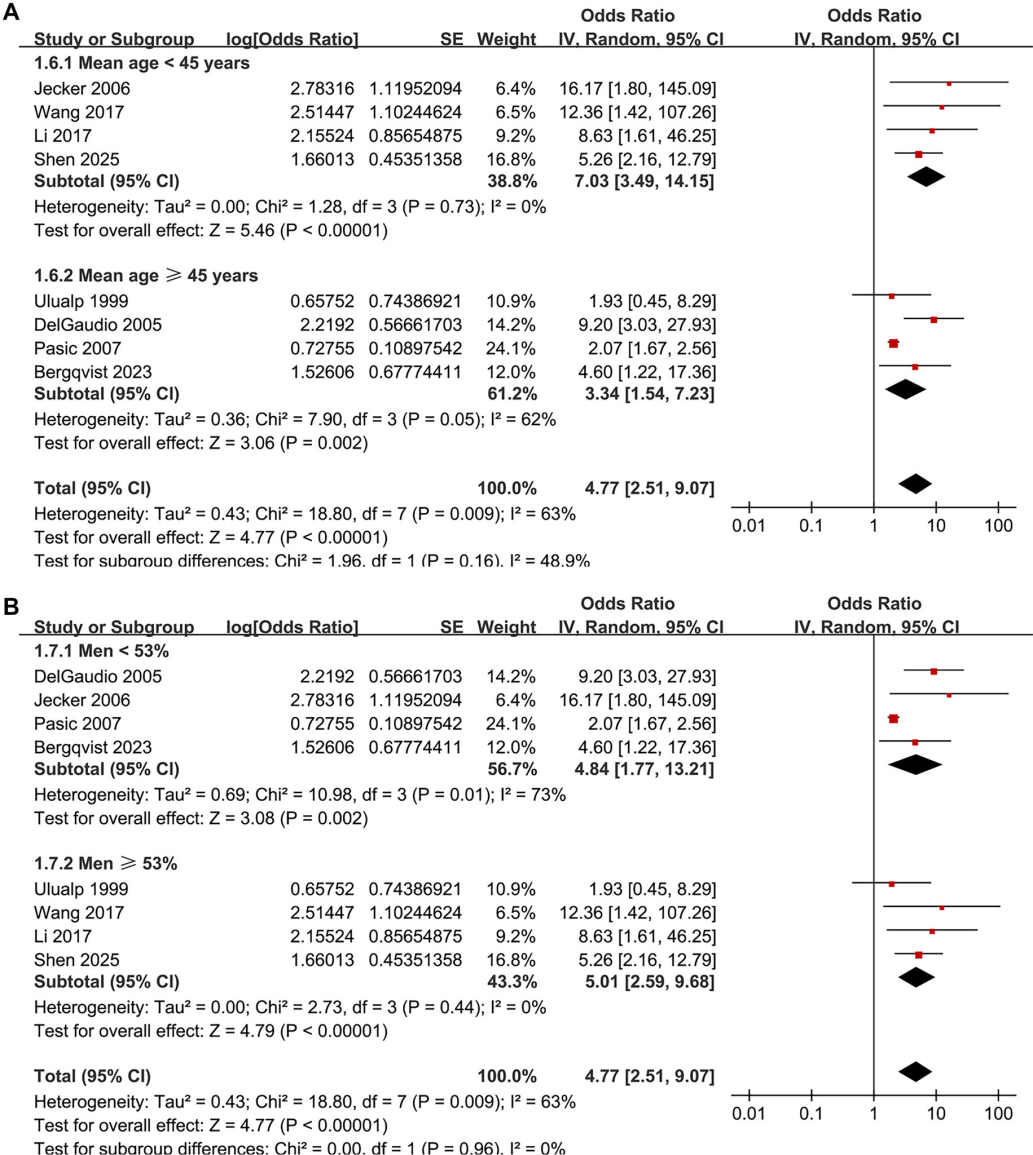
**Subgroup forest plots for the association between LPR and CRS in adults.** (A) Stratified by mean age (<45 vs ≥45 years): Pooled OR = 7.03 (95% CI 3.49–14.15) vs 3.34 (95% CI 1.54–7.23); *P* for subgroup difference = 0.16; (B) Stratified by proportion of men (<53% vs ≥53%): Pooled OR = 4.84 (95% CI 1.77–13.21) vs 5.01 (95% CI 2.59–9.68); *P* for subgroup difference = 0.96. Squares indicate study-specific ORs and diamonds pooled effects; *x*-axis is logarithmic with OR = 1 as the null. Abbreviations: LPR: Laryngopharyngeal reflux; CRS: Chronic rhinosinusitis; OR: Odds ratio; CI: Confidence interval.

**Figure 5.**
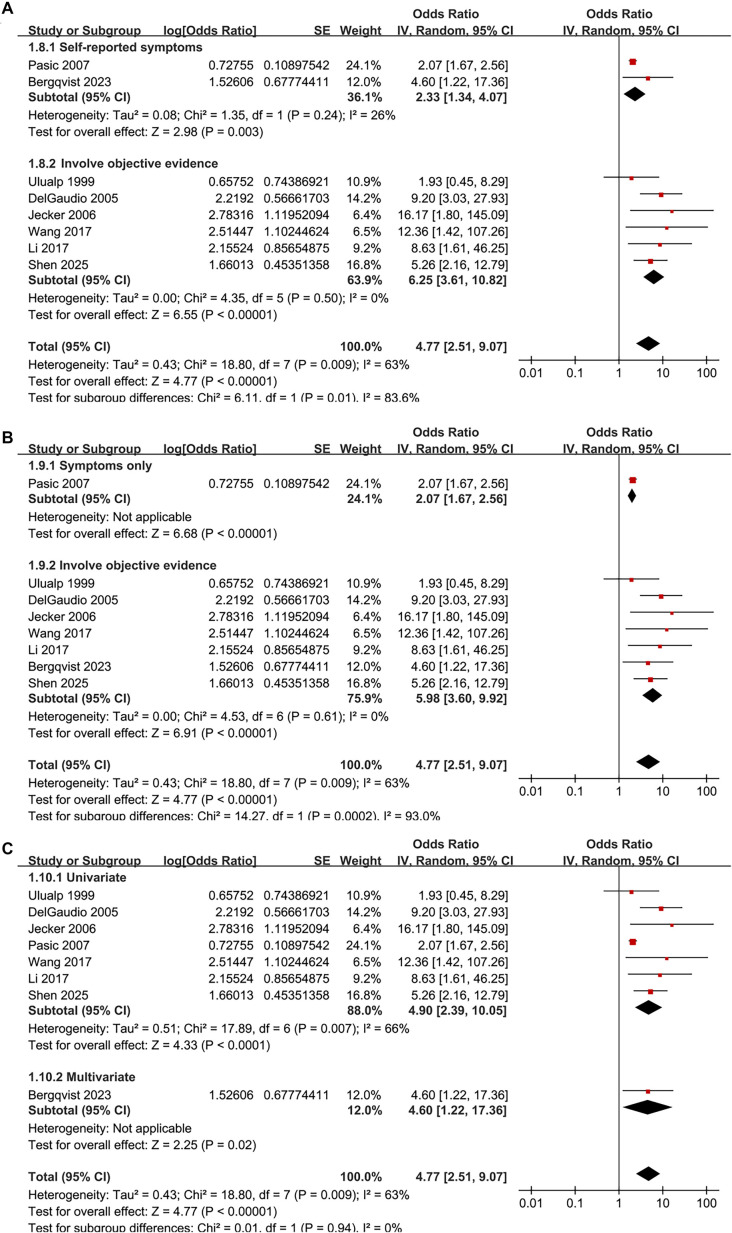
**Subgroup forest plots for the association between LPR and CRS in adults by diagnostic approach and analysis model.** (A) Stratified by LPR ascertainment: Studies using self-reported symptoms vs studies using objective evaluation; pooled OR = 2.33 (95% CI 1.34–4.07) vs 6.25 (95% CI 3.61–10.82), *P* for subgroup difference = 0.01; (B) Stratified by CRS definition: Symptoms only vs objective evidence; pooled OR = 2.07 (95% CI 1.67–2.56) vs 5.98 (95% CI 3.60–9.92), *P* for subgroup difference <0.001; (C) Stratified by analytic model: Univariate vs multivariate estimates; pooled OR = 4.90 (95% CI 2.39–10.05) vs 4.60 (95% CI 1.22–17.36), *P* for subgroup difference = 0.94. Squares indicate study-specific ORs with 95% CIs; diamonds indicate pooled effects; the *x*-axis is logarithmic with OR = 1 as the null. Abbreviations: LPR: Laryngopharyngeal reflux; CRS: Chronic rhinosinusitis; OR: Odds ratio; CI: Confidence interval.

The pooled results of the eight studies [14–21] indicate a significant association between LPR and an increased prevalence of CRS in adults (OR: 4.77, 95% CI: 2.51–9.07, *P* < 0.001; Figure 2A), with moderate heterogeneity (*P* for Cochrane *Q* test = 0.009, *I*^2^ ═ 63%, τ^2^ ═ 0.43, 95% PI: 1.13–20.07). Additionally, a sensitivity analysis employing the REML random-effects model with Hartung–Knapp adjustment yielded consistent results (OR: 4.59, 95% CI: 2.44–8.65, *P* < 0.001; *I*^2^ ═ 55.6%; Figure S1). Sensitivity analyses, which involved sequentially removing individual datasets, demonstrated stable results (OR: 4.11–5.98, *P* for all <0.05). Specifically, the sensitivity analysis focused on high-quality studies (NOS ≥ 7) showed consistent outcomes without significant heterogeneity (OR: 5.98, 95% CI: 3.60–9.92, *P* < 0.001; *I*^2^ ═ 0%; Figure 2B). Subgroup analyses revealed that results were consistent across studies from Western and Asian countries (OR: 3.96 vs 6.40, *P* for subgroup difference = 0.39; Figure 3A). Furthermore, subgroup analysis based on sample size indicated that studies with fewer than 100 participants reported a substantially larger association (OR: 10.16) compared to those with 100 or more participants (OR: 2.82), with a significant subgroup difference (*P* ═ 0.009), suggesting potential small-study effects (Figure 3B). The association did not differ significantly between patients with mean ages below or above 45 years (OR: 7.03 vs 3.34, *P* for subgroup difference = 0.16; Figure 4A) or between groups with men comprising less than or greater than 53% of the sample (OR: 4.84 vs 5.01, *P* for subgroup difference = 0.96; Figure 4B). A stronger association between LPR and CRS was observed in studies where LPR was diagnosed using objective assessments compared to those relying solely on self-reported symptoms (OR: 6.25 vs 2.33, *P* for subgroup difference = 0.01; Figure 5A), and in studies where CRS was diagnosed based on objective evidence compared to those based on symptoms alone (OR: 5.98 vs 2.07, *P* for subgroup difference < 0.001; Figure 5B). The analysis did not reveal significant differences between studies employing univariate and multivariate analyses (OR: 4.90 vs 4.60, *P* for subgroup difference = 0.94; Figure 5C).

### Publication bias

**Figure 6.**
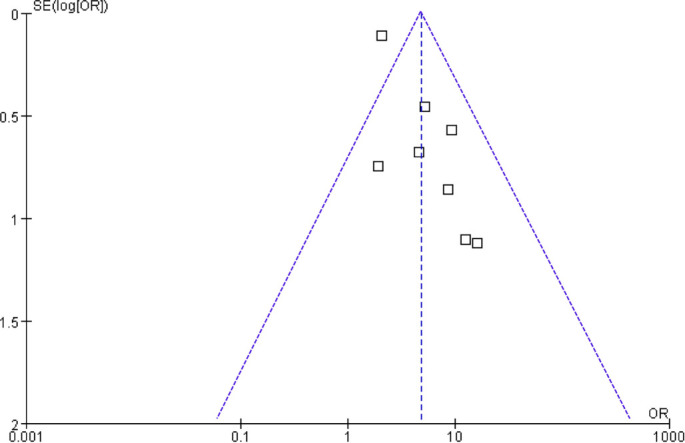
**Funnel plot assessing publication bias and small-study effects in the meta-analysis of the association between LPR and CRS.** The plot showed no clear asymmetry, and Egger’s test did not detect small-study effects (*P* ═ 0.35). Because only eight studies were included, visual and statistical assessments are underpowered and should be interpreted cautiously. Abbreviations: LPR: Laryngopharyngeal reflux; CRS: Chronic rhinosinusitis.

Funnel plots did not exhibit clear asymmetry, and Egger’s test did not provide statistical evidence of small-study effects (*P* ═ 0.35; Figure 6). However, due to the limited number of studies included, both visual inspection and Egger’s regression possess low statistical power, necessitating cautious interpretation of the absence of detected asymmetry.

## Discussion

This meta-analysis offers a comprehensive quantitative synthesis of the association between LPR and CRS in adults. By aggregating data from eight observational studies involving 3456 participants, we demonstrated a significant association between LPR and an increased prevalence of CRS. This relationship persisted in sensitivity analyses confined to high-quality studies and across most subgroups, underscoring the robustness of the findings. Notably, the strength of this association was greater when both LPR and CRS diagnoses were based on objective criteria, suggesting that methodological rigor and adequate sample size enhance the reliability of observed associations.

Recent contributions in the field provide context for these findings. A 2024 systematic review and meta-analysis by Aldajani et al. [30] examined a broad construct of “reflux diseases,” pooling studies of both GERD and LPR, and reported a significant overall association with CRS, as well as higher pH values and increased pepsin detection among CRS patients. Their work implies that reflux, when defined broadly, may be relevant to CRS pathophysiology; however, the heterogeneous exposure definitions, variability in diagnostic tools, and inclusion of therapeutic studies limited the ability to isolate the specific contribution of LPR [30]. Additionally, a Mendelian randomization analysis by Chen et al. [31] demonstrated that genetically predicted GERD increases the risk of CRS, supporting a potential causal role for esophageal reflux disease in CRS. However, Mendelian randomization instruments for LPR are not currently available, and GERD and LPR, while biologically related, represent clinically distinct phenotypes [31]. In this context, the present study provides a focused synthesis restricted to LPR, utilizing standardized prevalence-odds estimates and objective CRS definitions. By isolating LPR as the exposure of interest, our analysis clarifies its specific association with CRS, distinguishing it from GERD-based evidence and broader constructs of reflux disease.

The mechanisms by which LPR may contribute to the pathogenesis of CRS are biologically plausible and supported by both experimental and clinical evidence [32]. Refluxed gastric contents, particularly acid, pepsin, and bile salts, can reach the nasopharynx and paranasal sinuses [7, 33]. These substances disrupt epithelial barrier integrity, impair mucociliary clearance, induce pro-inflammatory cytokine production, and activate immune pathways in the sinonasal mucosa [34, 35]. Notably, pepsin remains enzymatically active even at neutral pH and has been detected in nasal secretions and mucosal tissues of CRS patients with LPR, indicating ongoing mucosal injury [36]. Additionally, reflux-induced edema and inflammation may alter sinonasal drainage pathways, promoting chronic stasis and increasing susceptibility to infection [37]. Although the cross-sectional design of most studies limits the ability to establish causality, these pathophysiological links provide a coherent explanation for the observed association.

Subgroup analyses offer further insights into how study characteristics may influence the observed relationship. The association between objective diagnostic methods for LPR, such as pH monitoring or pepsin detection, and higher effect estimates compared to symptom-based diagnoses emphasizes the importance of accurate exposure measurement. Symptom-based assessments of LPR are inherently subjective and prone to misclassification, potentially biasing associations toward the null. Similarly, the stronger association in studies that diagnosed CRS using objective measures, such as endoscopy or CT, compared with symptom-only diagnoses, underscores that rigorous case definitions enhance the detection of true relationships. These observations suggest that future research should employ standardized, validated, and objective diagnostic criteria for both LPR and CRS to minimize heterogeneity and improve comparability across studies. Interestingly, studies with smaller sample sizes (< 100) yielded significantly larger effect estimates than larger studies, which is more indicative of potential small-study effects—where smaller studies with variable methodologies or selective reporting tend to show exaggerated associations. This finding emphasizes the need for cautious interpretation and highlights the importance of adequately powered studies in future research. The lack of significant differences in associations across subgroups defined by mean participant age, sex distribution, or adjustment for confounders suggests that the relationship between LPR and CRS may be consistent across demographic strata and is not solely explained by basic confounding factors such as age and sex. However, residual confounding by other variables, including allergic sensitization, smoking, or comorbid conditions such as asthma and GERD, cannot be ruled out. Most included studies were cross-sectional and either unadjusted or adjusted for only a limited set of covariates, restricting the ability to account for these factors. This limitation should be considered when interpreting the results. The sensitivity analysis restricted to studies with higher methodological quality (NOS ≥ 7) not only confirmed the overall findings but also eliminated between-study heterogeneity, thereby strengthening confidence in the association. This suggests that some of the heterogeneity observed in the main analysis likely originated from methodological differences, including diagnostic definitions, participant selection, and confounding control. These findings underscore the importance of rigorous study design and reporting in future research to enhance evidence quality.

This meta-analysis possesses several notable strengths. First, it represents the most comprehensive and updated synthesis of the literature, incorporating studies from both Western and Asian countries and including recent research up to 2025. Second, we adhered to a prespecified protocol and followed PRISMA 2020 and Cochrane guidelines to ensure methodological transparency. To enhance robustness, given the limited number of studies, we supplemented the DL analysis with an REML–HKSJ model, which produced effect estimates with overlapping CIs, thereby supporting the stability of the primary findings. However, the wide PI underscores uncertainty regarding the magnitude of the association across different populations. Additionally, due to the limited number of eligible studies, our multiple dichotomized subgroup analyses should be interpreted cautiously. A meta-regression would have been methodologically preferable, but was not feasible because of the small number of studies and the absence of individual participant data. Finally, multiple sensitivity and subgroup analyses were conducted, and the consistent direction of results across these analyses enhances the robustness of the findings.

Nonetheless, several limitations warrant cautious interpretation. The primary limitation is the cross-sectional design of all included studies, which precludes inferences about temporal or causal relationships between LPR and CRS. It remains unclear whether LPR contributes to the initiation of CRS or whether CRS exacerbates LPR through nasal obstruction and increased negative intrathoracic pressure [38]. Prospective cohort studies or interventional studies targeting LPR would be valuable for clarifying causality. Second, heterogeneity in diagnostic criteria for both LPR and CRS across studies may have introduced misclassification and affected effect estimates. Although subgroup analyses based on diagnostic methods illuminate this issue, the lack of uniform gold-standard definitions limits the comparability of existing studies. Furthermore, while distinguishing between CRS phenotypes—specifically CRS with nasal polyps (CRSwNP) and CRS without nasal polyps (CRSsNP)—is clinically significant, most of the studies included did not provide separate effect estimates for these subgroups. This limitation precludes the possibility of conducting a phenotype-specific meta-analysis. Future studies with consistent stratification are necessary to determine whether the association between LPR and CRS differs by polyp status. Third, most studies lacked comprehensive adjustment for potential confounders beyond age and sex, raising the possibility of residual confounding. Fourth, the relatively small number of available studies limited the power to explore more nuanced subgroup effects, such as the influence of comorbid allergic rhinitis, asthma, or GERD. Moreover, data from longitudinal or interventional trials were not available, leaving the clinical significance of reducing LPR for the prevention or management of CRS uncertain. Finally, although neither the funnel plot nor Egger’s test indicated statistical evidence of publication bias, the power of these methods is limited when fewer than ten studies are available. Furthermore, the pattern observed in the sample-size subgroup—where smaller studies reported larger effect estimates—suggests the possibility of small-study effects arising from selective reporting, methodological variability, or chance. These considerations reinforce the need for cautious interpretation.

From a clinical perspective, these findings suggest that clinicians should be aware of the potential link between LPR and CRS, particularly in patients with persistent or refractory CRS. Given that all included studies were cross-sectional, the pooled effect represents differences in the prevalence odds of CRS rather than incidence or risk. Consequently, the findings indicate an association rather than a temporal or causal relationship. Furthermore, as almost all studies reported unadjusted ORs, the results should not be interpreted as independent of confounding factors. The observed associations may partly reflect shared risk factors or residual confounding. Future research should focus on prospective longitudinal studies to determine whether LPR precedes CRS onset and whether effective management of LPR reduces the risk or severity of CRS. Standardization of diagnostic definitions and the use of objective methods for both LPR and CRS will be crucial for improving comparability and reducing heterogeneity. Additionally, mechanistic studies exploring the role of pepsin and other reflux components in sinonasal mucosal inflammation could provide further biological insights. RCTs of anti-reflux interventions in patients with CRS who have objectively confirmed LPR would be particularly informative for establishing causality and assessing potential therapeutic benefits. For example, a clinical trial in 2018 by Anzić et al. [39] demonstrated that 8 weeks of omeprazole 20 mg once daily significantly reduced both LPR and CRS symptom and endoscopic scores compared with placebo, despite most patients having residual disease at the end of treatment. Future trials could build on this by testing higher or guideline-recommended proton pump inhibitor (PPI) doses and longer treatment durations, evaluating combined medical and lifestyle anti-reflux interventions, or comparing pharmacologic therapy with alternatives such as alginate formulations or surgical reflux control. These studies should also utilize standardized CRS outcomes—such as validated symptom scores, endoscopic grading, and imaging-based assessments—and include long-term follow-up to determine whether controlling LPR reduces CRS recurrence, improves quality of life, and lessens the need for surgical management.

## Conclusion

In summary, this meta-analysis suggests that adults with LPR have higher prevalence odds of CRS compared to those without LPR. Given the cross-sectional and predominantly unadjusted nature of the included evidence, the findings should be interpreted as associational rather than causal. Prospective, well-controlled studies are needed to determine temporality and clarify whether addressing LPR influences CRS outcomes.

## Supplemental data

Supplemental data are available at the following link: https://www.bjbms.org/ojs/index.php/bjbms/article/view/13354/4096.

## Data Availability

All data generated or analyzed during this study are included in this published article.
